# Analysis of circulating tumour cell and the epithelial mesenchymal transition (EMT) status during eribulin-based treatment in 22 patients with metastatic breast cancer: a pilot study

**DOI:** 10.1186/s12967-018-1663-8

**Published:** 2018-10-20

**Authors:** Yoshiya Horimoto, Emi Tokuda, Fumi Murakami, Toshitaka Uomori, Takanori Himuro, Katsuya Nakai, Gotaro Orihata, Kotaro Iijima, Shinsaku Togo, Hideo Shimizu, Mitsue Saito

**Affiliations:** 1 Department of Breast Oncology, Juntendo University School of Medicine, 2-1-1 Hongo, Bunkyo-ku, Tokyo, 113-0033 Japan; 2 Department of Pathology and Oncology, Juntendo University School of Medicine, 2-1-1 Hongo, Bunkyo-ku, Tokyo, 113-0033 Japan; 3 Department of Respiratory Medicine, Juntendo University School of Medicine, 2-1-1 Hongo, Bunkyo-ku, Tokyo, 113-0033 Japan

**Keywords:** Breast cancer, Circulating tumour cell, Liquid biopsy, Eribulin, Epithelial mesenchymal transition

## Abstract

**Background:**

Liquid biopsy approaches, such as measuring circulating tumour cells (CTCs), have recently been introduced in several clinical studies. However, the development of CTCs as a predictive marker for treatment effects on breast cancer remains an enormous task. We investigated CTCs, including epithelial mesenchymal transition (EMT) status, from metastatic breast cancer patients who had received eribulin-based treatment, which reportedly suppresses EMT as a means of tumour suppression. Our aim was to test the possibility of this method serving as a tool predicting eribulin efficacy.

**Methods:**

Twenty-two patients were enrolled and peripheral blood samples were collected before eribulin treatment. CTCs were then examined using a Microfluidic Chip device. CTCs positive for vimentin and pan-cytokeratin were defined as mesenchymal and epithelial CTCs, respectively. Progression-free survival (PFS) and clinical response were assessable in 20 and 18 patients, respectively, in relation to the number of CTCs.

**Results:**

Numbers of total CTCs were significantly increased in patients with progressive disease during treatment (p = 0.006). Median PFS was 14.6 weeks and patients with more total and mesenchymal CTCs at baseline had significantly shorter PFS (p = 0.0013 and 0.013, respectively). Multivariate logistic regression analysis revealed small number of total baseline CTCs and long disease-free survival to be related to long PFS (p = 0.0004 and 0.020, respectively).

**Conclusions:**

Our data suggest that determining both mesenchymal and epithelial CTCs at baseline might be a good tool for predicting eribulin responsiveness. Evaluation of mesenchymal CTC can be considered as a parameter in larger studies, while most clinical trials are currently employing only the detection of the epithelial cellular adhesion molecule (EpCAM).

**Electronic supplementary material:**

The online version of this article (10.1186/s12967-018-1663-8) contains supplementary material, which is available to authorized users.

## Background

### CTC analysis in breast cancer research

Liquid biopsy approaches, such as measuring circulating tumour cells (CTCs) and DNA, have recently been introduced in clinical studies on a broad range of cancer types. Several studies have shown high numbers of CTCs to correlate with poorer outcomes in patients with both early and metastatic breast cancer (MBC), such that CTCs are now a relatively well established prognostic marker [1–3]. Moreover, two studies revealed some patients to have HER2 positive CTCs, despite their primary tumours being HER2-negative [4, 5] and such information might aid in making treatment decisions. However, there are still many problems, including standardisation of analysis since laboratories employ different techniques, before CTC analysis can be introduced into routine clinical practice. Also, evidence supporting CTCs as a marker predicting treatment effects in breast cancer remains insufficient [2, 4, 6].

### Improvement of technology in CTC analysis

The CellSearch^®^ System has become the most widely used technique for CTC analysis in both pre-clinical and clinical studies [7]. This system captures CTCs using antibodies against epithelial cellular adhesion molecule (EpCAM) and several epithelial cell surface markers, including CK8, 18 and 19. EpCAM is a conventional marker well-known to be expressed in epithelial-origin cancer cells. However, a growing body of evidence has revealed that EpCAM is not expressed in all CTCs [8–10]. A subpopulation of CTCs with decreased levels of epithelial markers might escape from EpCAM-based detection [8] and epithelial-to-mesenchymal transition (EMT) and could thereby be a major reason for this phenomenon [6, 8, 11–15].

To overcome this defect in EpCAM-based detection, technologies for CTC analysis have advanced and the EMT status of CTCs can now also be assessed [14, 16, 17]. For instance, changes in EMT status during treatments of individual patients with MBC were reported [6]. Several studies have shown improved CTC detection by employing EMT markers in addition to epithelial markers [18, 19].

### Eribulin treatment in MBC patients

MBC is essentially incurable although many treatment options including endocrine agents and chemotherapy are available. Individual MBCs respond differently even to the same treatments. Therefore, we often encounter difficulties in choosing the most appropriate treatments with optimal timing for individual patients. Thus, predictive markers for therapeutic efficacy are eagerly awaited.

Eribulin is a microtubule-depolymerising agent and was introduced as an option for MBC patients, following confirmation of clinically meaningful improvement in overall survival [20]. Eribulin was also recently approved by the Food and Drug Administration of the United States as a liposarcoma treatment. Interestingly, the suppression of EMT might reportedly be the mechanism by which eribulin regulates breast cancer progression both in vitro and in vivo [21, 22].

We investigated CTCs in MBC patients who had received eribulin, employing an established system that can evaluate EMT status. The current study aimed to test the possibility of this method serving as a tool for predicting eribulin efficacy. We are also interested in whether the EMT status of CTCs is suppressed in response to eribulin treatment.

## Methods

### Patients and treatments

Twenty-two patients with metastatic/stage IV breast cancer, who had started eribulin-based treatments at our department during the January through December 2017 period were enrolled. Seventeen patients developed metastatic disease after undergoing curative surgery for primary breast cancer, while 5 had Stage IV disease. Clinicopathological features of the 22 patients are shown in Table 1. Mean patient age at the time of starting eribulin was 57 (range 38–81) years. The intrinsic subtype rates of the primary tumours were: luminal-HER2(−) 64%, luminal-HER2(+) 9%, HER2 type 5%, and triple negative (TN) 23%. Median disease-free-survival after curative surgery was 78 months (range 12–125). Metastatic sites were bone (59%), liver (36%), the lungs (32%), pleura (9%), brain (9%) and others (9%). Eribulin was administrated as the first, second, third/more line of chemotherapy for metastatic disease in 36%, 55% and 9% of patients, respectively.

**Table 1:** Clinicopathological features of 22 patients

n	22
Median age^a^ (range)	57 (38–81)
Characteristics of primary tumour	
Histology	
IDC (NST)	18 (82%)
Special type	4 (18%)
Grade	
High	5 (23%)
Low-intermediate	13 (59%)
Unknown	4 (18%)
Intrinsic subtype^b^	
Luminal-HER2(−)	13 (59%)
Luminal-HER2(+)	3 (14%)
HER2	1 (5%)
Triple negative	5 (23%)
DFS (months)^c^ (range)	78 (12–125)
Metastatic site	
Bone	13 (59%)
Liver	8 (36%)
Lungs	7 (32%)
Pleura	2 (9%)
Brain	2 (9%)
Others	2 (9%)
Number of previous chemotherapies for metastatic disease	
0	8 (36%)
1–2	12 (55%)
3−	2 (9%)

*DFS* disease-free survival, *IDC* invasive ductal carcinoma, *NST* non-special type

^a^At the time of starting eribulin

^b^HER2 overexpression was defined as IHC (3+) or FISH (+)

^c^For 17 patients who underwent curable surgery for the primary tumour

Eribulin mesylate was administered at the standard dose of 1.4 mg/m^2^ on day 1 and day 8. During treatment, drug doses were gradually reduced as needed, such as in cases with neutropenia. Anti-HER2 drugs, such as Trastuzumab and Pertuzumab, were simultaneously administered according to the HER2 status of the patient’s tumour.

Peripheral blood (10 ml) samples were collected before eribulin treatment. The samples were sent to the Nihon Gene Research Laboratories (Japan) within 24 h for analysis of CTCs. CTCs were re-examined in some patients when treatment effects were evaluated (details presented in the CTC analysis section). This study was carried out with approval from the ethics committee of Juntendo University Hospital (no: 16-139) and all samples were collected after obtaining written informed consent from the patients.

### Evaluations of clinical response and progression free survival (PFS)

**Fig. 1:**
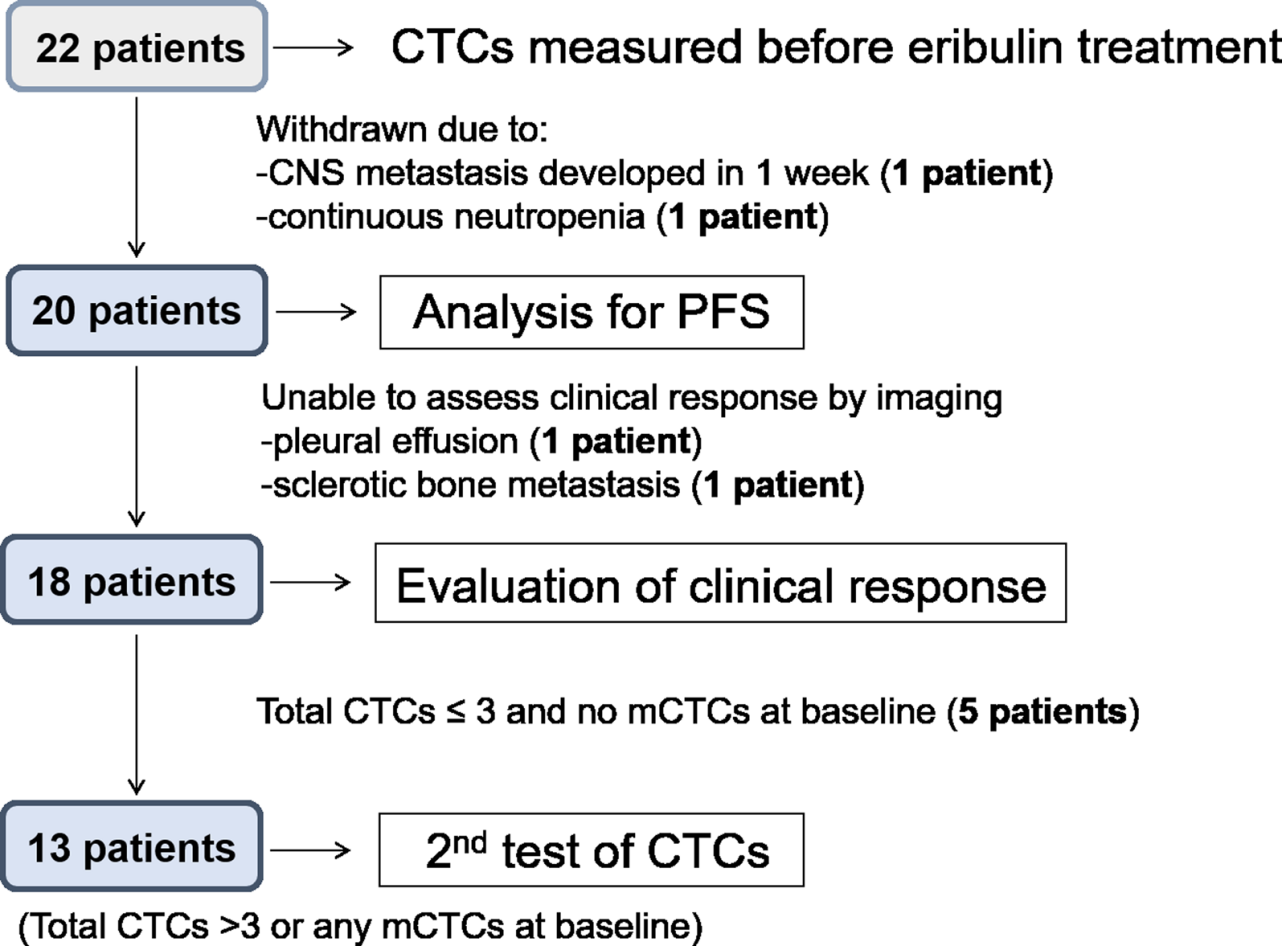
Flow chart of analysis criteria. PFS was analysed in 20 patients, after 2 had been withdrawn, due to rapid development of CNS metastasis and continuous neutropenia. Of these 20 patients, we were able to evaluate clinical responses based on imaging in 18. Thirteen patients, with at least 4 total CTCs or any mCTCs at baseline, underwent a second CTC test when treatment effects were assessed

Clinical responses were evaluated with radiographic images based on the RECIST (version 1.1) guideline [23], which defines complete response (CR), partial response (PR), stable disease (SD), and progressive disease (PD). Analysis criteria are listed in Fig. 1. Following the baseline CTC assessment, 2 patients were withdrawn and did not undergo evaluation of treatment effects: 1 patient developed metastasis involving the central nerve system just 1 week after the first eribulin administration and the other patient suffered continuous bone marrow suppression. Evaluations were basically conducted after three treatment courses but the timing differed among cases, since it was determined based on individual disease states. One patient had only non-measurable metastatic disease, i.e. pleural effusion. Another patient had only bone metastases appearing as sclerotic changes on computed tomography scans. Thus, we excluded these 2 patients from the evaluation of clinical response but they were included in the population for PFS analysis.

### CTC analysis

CTCs were examined using a Microfluidic Chip device at Nihon Gene Research Laboratories (Japan). This commercially available system captures and isolates rare CTCs from blood samples with 56,320 wells, based on their size and deformability differences from blood cells [16]. Thereby, this system provides very high efficiency. Fluorescence images of cells, including EMT markers, can be obtained using an automated staining and scanning system.

**Fig. 2:**
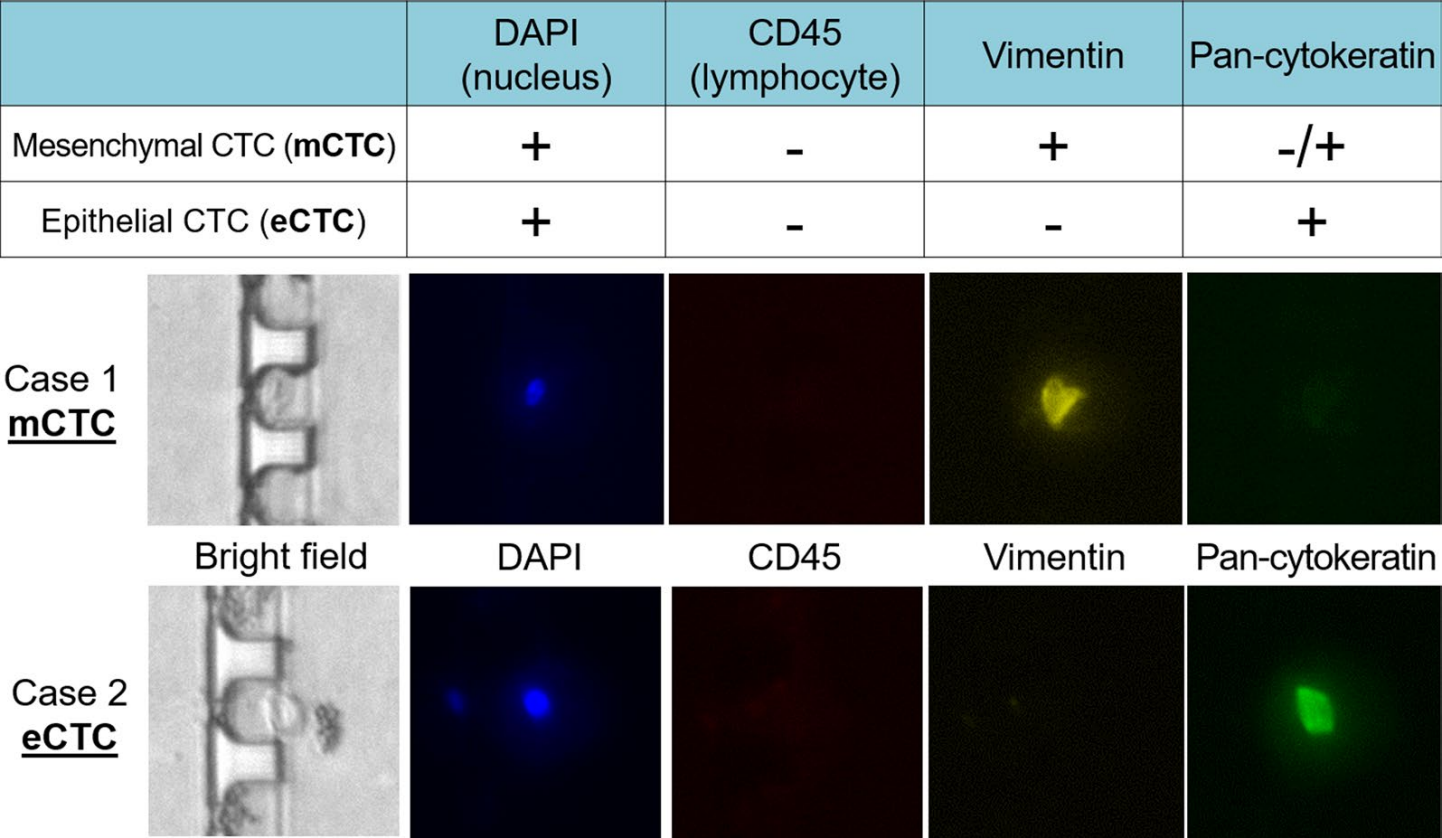
CTC detection and mCTC and eCTC definitions. CTCs captured and isolated from blood samples were stained and scanned automatically. Representative fluorescence images of mCTC-predominant and eCTC-predominant cases in our cohort are shown

CTCs positive for vimentin and pan-cytokeratin were defined as mesenchymal (mCTCs) and epithelial (eCTCs), respectively. Representative mCTC and eCTC cases in our cohort are shown in Fig. 2. Case 1 is positive only for Vimentin and was thus judged as having mCTC. Case 2 is positive for cytokeratin and was thus defined as having eCTC. CTCs positive for both cytokeratin and vimentin, observed in a few cases, were defined as mCTCs in the current study, as indicated in Fig. 2. Patients, who had at least four CTCs per 4 ml in total or any mCTCs at the beginning of the treatment, underwent the second CTC test when treatment effects were evaluated.

### Statistical analysis

Statistical analyses were performed using JMP 11.2.1 statistical software (SAS Institute Inc., Cary, NC, USA). Associations between clinicopathological parameters and patient outcomes were evaluated using Fisher’s exact test. For comparisons of the median values of changes in the numbers of CTCs during treatments, examinations of paired data were carried out employing the Mann–Whitney U test. Kaplan–Meier curves were estimated and the log-rank test was applied for comparisons of the survival distributions of two populations. The logistic regression model was constructed using the stepwise procedure with the minimal Bayesian information criterion in an attempt to identify predictors of long PFS. Among factors selected by univariate analysis, DFS and total CTCs were selected as full-model variables for the present analysis. A p < 0.05 was considered to indicate a statistically significant difference.

## Results

### mCTCs were frequently observed in patients with TN MBC

CTCs were detected in 21 of 22 patients. The median number of total CTCs was 3.0 per 4.0 ml. When the results were analysed according to intrinsic subtype, there were no differences in total number of CTCs (Additional file 1: Table S1). However, mCTCs were more commonly observed in TN than in other cancer cases and the mCTC rate was significantly higher in TN cases than in those with luminal HER2-negative MBC (p < 0.05).

### PD group tended to have more total and mCTCs

**Fig. 3:**
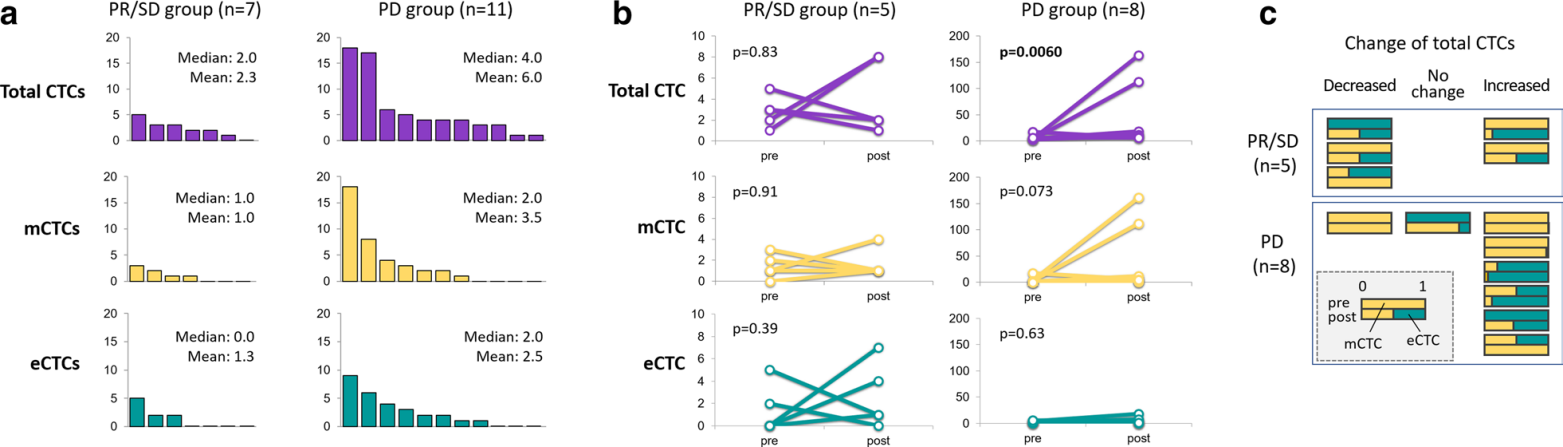
Baseline CTCs and changes in CTCs during treatments according to treatment effects. **a** Bar charts show details of baseline CTCs according to treatment responses in 18 patients. Total CTCs, mCTCs and eCTCs are indicated in purple, yellow and green, respectively. **b** Line graphs indicate change in CTCs during eribulin administration according to treatment responses in 15 patients, all of whom received a second CTC test. **c** Proportions of mCTC and eCTC in individual patients during eribulin administration, according to changes in total CTCs, are shown. As indicated in the grey square, the upper bar shows baseline status, the lower bar that during treatment. Yellow and green bars represent the rates of mCTC and eCTC, respectively

Treatment responses in 18 patients were analysed. Rates of PR, SD and PD were 17% (3 cases), 22% (4) and 61% (11), respectively, while there were no CR cases. Clinical benefits, i.e. PR and SD lasting longer than 3 months, were obtained in 7 patients (39%). When the patients were categorised into two groups, PR/SD and PD, total and mCTC were higher in the PD group (mean 2.3 vs 6.0 and 1.0 vs 3.5, respectively), although the differences were not statistically significant (Fig. 3a).

### Total CTCs increased during treatments in the PD group

Changes in CTCs during treatments were evaluated in 13 patients who underwent the second CTC test. As shown in Fig. 3b, the total CTC number was significantly increased in the PD group (p = 0.0060), with mCTC showing a similar tendency (p = 0.073). However, in the PR/SD cases, no trends were observed across all CTC types.

Furthermore, we investigated changes in the proportions of mCTC and eCTC during treatments. The results, according to clinical response, are shown in Fig. 3c. No significant trend was observed in the mCTC rate, regardless of the change in the pattern of total CTCs. Interestingly, when we focus on patients whose CTCs at baseline were all mCTC (3 patients each in the PR/SD and PD groups), all 3 in the PR/SD group showed a decrease in the mCTC rate, while none of the 3 in the PD group showed a decrease.

### Patients with more total CTCs and mCTCs had shorter PFS

**Fig. 4:**
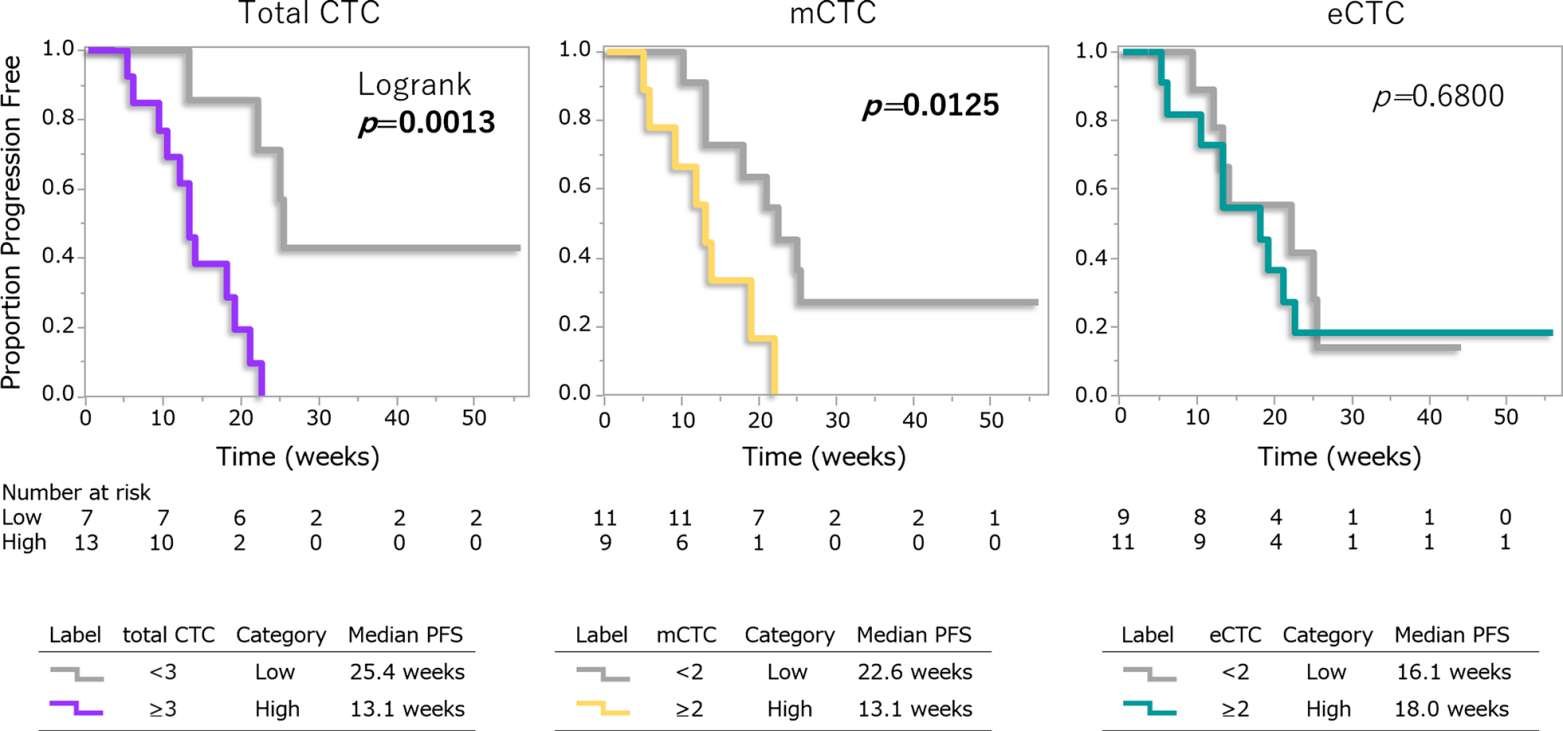
Kaplan–Meier curves of PFS according to CTC types. Kaplan–Meier curves for PFS in 20 patients according to each CTC type are shown. The log-rank test was applied for comparisons of the survival distributions of the two groups

PFS was assessed in 20 patients. Median PFS was 14.6 weeks and 3 patients are still receiving treatments. Kaplan–Meier curves for PFS according to CTC types are presented in Fig. 4. Patients with more total CTCs had significantly shorter DFS than those with fewer CTCs (p = 0.0013). The same trend was observed for mCTCs with a statistically significant difference (p = 0.013). On the other hand, there were no differences according to numbers of eCTCs. When the differences between the total and mCTC groups were compared, the gap was much wider for total CTC than for mCTCs.

### Small number of total CTCs and long DFS predict long PFS

Finally, we sought factors possibly predicting long PFS (6 months or longer). Fisher’s exact test revealed that total CTCs (less than 3), mCTCs (less than 2) and DFS (60 months or more) correlated with long PFS, as well as other clinicopathological factors such as age, tumour grade, ER and HER2 status, metastatic site (visceral vs non-visceral), and previous chemotherapy (Additional file 2: Table S2). We then employed a multivariate logistic regression model for total CTCs and DFS, selected by the stepwise procedure. Both total CTCs and DFS were found to be significantly and independently related to long PFS (p = 0.0004 and 0.020, respectively) (Table 2).

**Table 2:** Logistic model for predicting long PFS

Variables	OR (CI)	p-value
Total CTCs (< 3 vs 3 or more)	6.67E+14 (17.4 − ∞)	*0.0004*
DFS (60 months or more vs < 60)	2.32E+14 (2.78 − ∞)	*0.020*

Italic values indicate significance of p value (*p* < 0.05)

*OR* odds ratio, *CI* confidence interval

## Discussion

In the current study, we found that total CTCs, comprised of mCTCs and eCTCs, might predict eribulin efficacy. To our knowledge, the current study is the first to examine the possibility of baseline CTCs serving as a predictive marker specific for eribulin-based treatment. We also monitored changes in the number and EMT status of CTCs during eribulin administration.

Changes in CTC numbers have previously been demonstrated in several studies focusing on other treatments [6, 24, 25]. Yu et al. monitored 11 MBC patients and total CTCs were decreased in responders to various chemotherapeutic regimens [6]. Likewise, in a randomised phase III trial involving 56 MBC patients, a population who showed early reductions in CTCs (detected with the CellSearch System) during chemotherapy had longer PFS [25]. It is understandable that the number of CTCs might simply reflect the total tumour burden in a MBC patient. Our data showed similar trends during eribulin treatment. An increase in CTC numbers was observed in the PD group, regardless of CTC types.

According to several reports, one of the mechanisms underlying the anti-tumour effects of eribulin might be suppression of EMT [21, 22]. Changes in E-cadherin subcellular localisation and reversal of EMT were demonstrated in response to eribulin in breast cancer cell lines [21]. Yoshida et al. showed that eribulin reversed the EMT phenotype in an in vivo model, resulting in suppression of tumour metastasis [22]. Thus, we initially expected that mCTCs would be decreased in responders to eribulin. Indeed, in the 6 patients whose CTCs were 100% mCTCs at baseline, the mCTC rate was decreased in the three with PR/SD (Fig. 3c), while the three with PD showed no changes. However, overall, no changes or even trends were observed in either the absolute number or the rate of mCTCs rising and decreasing in the PR/SD group (Fig. 3b, c). Therefore, based on our data, we could not reach a conclusion regarding this issue.

Whether regulation of EMT in CTCs contributes to patient outcomes is still unknown since the opposite phenomenon, MET (mesenchymal epithelial transition), is considered to occur when CTCs develop homing to a distant organ [26]. Further studies are clearly needed to examine this issue in detail.

Patients with more total CTCs and mCTCs had shorter PFS. Our data correlate with those of previous reports showing elevated baseline CTCs to be a predictive marker for poor outcomes after a variety of chemotherapeutic regimens, although most such large trials employed EpCAM-based detection [2, 24, 25, 27]. Our data clearly showed no differences in PFS according to eCTC number (Fig. 4). Whether the observed difference is due to eribulin treatment is as yet unknown.

Meanwhile, patients with more total CTCs and mCTCs had significantly shorter DFS than those with fewer CTCs. When the differences in PFS between the two groups, based on total and mCTCs, were compared, the gap was much wider for total CTCs (Fig. 4). This observation suggests that measuring mCTCs only is not sufficient to predict PFS. Instead, assessing total CTCs, based on mCTC and eCTC, might be the optimal approach.

We believe that assessing mCTCs, in addition to eCTCs, would improve the current EpCAM-based analysis, allowing it to serve as a tool for assessing patient outcome and treatment effects.

Limitations of the current study were lack of a control group and the small number of cases. Thus, as a next step, our approach of assessing mCTCs and eCTCs should be introduced in randomised prospective studies, comparing eribulin with other forms of chemotherapy, with a large number of patients.

## Conclusions

Our data suggest that determinations of both mCTCs and eCTCs at baseline provide a good tool for predicting eribulin responsiveness and our observations also suggest that adding mCTC evaluation to the treatment selection process for MBC might merit examination in future studies. In summary, this approach may also be worth incorporating into ongoing large trials mostly employing EpCAM-based methods only.

## Additional files

**Additional file 1: Table S1.** Number of CTCs according to intrinsic subtype.

**Additional file 2: Table S2.** Univariate analysis of factors related to long PFS.

## Abbreviations

CTCs: circulating tumour cells
CR: complete response
DFS: disease-free survival
eCTCs: epithelial circulating tumour cells
EMT: epithelial mesenchymal transition
IDC: invasive ductal carcinoma
MBC: metastatic breast cancer
mCTCs: mesenchymal circulating tumour cells
NST: non-special type
PD: progressive disease
PFS: progression-free survival
PR: partial response
SD: stable disease
TN: triple negative
