# Supplementary material for: Analysis of circulating tumour cell and the epithelial mesenchymal transition (EMT) status during eribulin-based treatment in 22 patients with metastatic breast cancer: a pilot study
Source: J Transl Med. 2018 Oct 20;16:287. doi: 10.1186/s12967-018-1663-8 (PMC6195982; doi:10.1186/s12967-018-1663-8)
Supplement: Supplementary file 1 — Additional file 1: Table S1. Number of CTCs according to intrinsic subtype. [file 12967_2018_1663_MOESM1_ESM.pdf]

Table S1. Number of CTCs according to intrinsic subtype

Intrinsic subtype	n	Number of total CTCs (range)*	Number of mCTCs (range)*	Proportion of mCTCs*
Luminal-HER2(-)	13	3.0 (0-18)	0.0 (0-18)	10%**
Luminal-HER2(+)	3	3.0 (2-3)	1.0 (0-3)	33%
HER2	1	1	0	0%
Triple negative	5	4.0 (1-17)	3.0 (1-8)	100%**

*median values, **p<0.05
