# Supplementary material for: Analysis of circulating tumour cell and the epithelial mesenchymal transition (EMT) status during eribulin-based treatment in 22 patients with metastatic breast cancer: a pilot study
Source: J Transl Med. 2018 Oct 20;16:287. doi: 10.1186/s12967-018-1663-8 (PMC6195982; doi:10.1186/s12967-018-1663-8)
Supplement: Supplementary file 2 — Additional file 2: Table S2. Univariate analysis of factors related to long PFS. [file 12967_2018_1663_MOESM2_ESM.pdf]

Table S2. Univariate analysis of factors related to long PFS

		PFS≥6mos	PFS<6mos	P-value
n		5	15	
Age				n.s.
	55 or less	2	7	
	>55	3	8	
Histology				n.s.
	IDC (NST)	4	9	
	Others	1	6	
Grade				n.s.
	High	1	4	
	Low	3	8	
	Unknown	1	3	
Ly				n.s.
	Positive	5	4	
	Negative	0	5	
	Unknown	0	6	
ER				n.s.
	Positive	3	11	
	Negative	2	4	
PgR				n.s.
	Positive	3	10	
	Negative	2	5	
HER2				n.s.
	Positive	2	1	
	Negative	3	14	
StageIV				n.s.
	Yes	0	5	
	No	5	10	
DFS (months)				**0.044**
	60 or longer	5	4	
	<60	0	6	
Visceral metastasis				n.s.
	Yes	3	12	
	No	2	3	
Total CTCs				**0.0014**
	3 or more	0	13	
	<3	5	2	
mCTC				**0.038**
	2 or more	0	9	
	<2	5	6	
eCTC				n.s.
	2 or more	2	9	
	<2	3	6	
Eribulin as 1st chemo				n.s.
	Yes	2	5	
	No	3	10	
